# Frailty in Aging and the Search for the Optimal Biomarker: A Review

**DOI:** 10.3390/biomedicines10061426

**Published:** 2022-06-16

**Authors:** Magdalena Sepúlveda, Diego Arauna, Francisco García, Cecilia Albala, Iván Palomo, Eduardo Fuentes

**Affiliations:** 1 Thrombosis Research Center, Medical Technology School, Faculty of Health Sciences, Interuniversity Center for Healthy Aging, Universidad de Talca, Talca 3480094, Chile; magdalena.sepulveda@utalca.cl (M.S.); darauna@utalca.cl (D.A.); 2 Department of Geriatric Medicine, Complejo Hospitalario de Toledo, 45007 Toledo, Spain; franjogarcia@icloud.com; 3 Unidad de Nutrición Pública, Instituto de Nutrición y Tecnología de los Alimentos, Interuniversity Center for Healthy Aging, Universidad de Chile, Santiago 8320000, Chile; calbala@uchile.cl

**Keywords:** frailty, aging, biomarkers, older people, diagnosis

## Abstract

In the context of accelerated aging of the population worldwide, frailty has emerged as one of the main risk factors that can lead to loss of self-sufficiency in older people. This syndrome is defined as a reduced state of physiological reserve and functional capacity. The main diagnostic tools for frailty are based on scales that show deficits compared to their clinical application, such as the Fried frailty phenotype, among others. In this context, it is important to have one or more biomarkers with clinical applicability that can objectively and precisely determine the degree or risk of frailty in older people. The objective of this review was to analyze the biomarkers associated with frailty, classified according to the pathophysiological components of this syndrome (inflammation, coagulation, antioxidants, and liver function, among others). The evidence demonstrates that biomarkers associated with inflammation, oxidative stress, skeletal/cardiac muscle function, and platelet function represent the most promising markers of frailty due to their pathophysiological association with this syndrome. To a lesser extent but with the possibility of greater innovation, biomarkers associated with growth factors, vitamins, amino acids, and miRNAs represent alternatives as markers of this geriatric syndrome. Likewise, the incorporation of artificial intelligence represents an interesting approach to strengthening the diagnosis of frailty by biomarkers.

## 1. Introduction

The world’s population is aging rapidly [1]. Twelve percent of the world’s population is 60 years old or older, and by mid-century, this population could reach 21.5% [2]. During this same period, the population aged ≥ 80 years would increase from 1.7% to 4.5% [2]. According to the World Health Organization (WHO), healthy aging is a process of development and maintenance of functional capacity that allows well-being in old age [3]. Concerning this, frailty emerges as one of the main risk factors that can lead older people to lose self-sufficiency, diminishing their quality of life and health [4]. Frailty is defined as a clinically recognizable state in which older people’s ability to cope with everyday stressors is compromised by an increased vulnerability, which is caused by reduced physiological reserve and decline in multiple physiological systems, all of the above associated with age [5]. The importance of frailty lies in the fact that frail older people have a higher risk of premature death, in addition to numerous adverse health outcomes, such as falls, fractures, dementia, disability, impaired quality of life, and excessive use of state health resources [6,7,8,9,10,11,12,13,14]. In this sense, the WHO developed the ICOPE guide (Integrated Care for Older People) with the aim of promoting, improving, or maintaining both the intrinsic and functional capacity of older persons to achieve healthy aging, being a key point in the prevention of frailty and a correct diagnosis through tools and/or biomarkers [15,16,17].

There are currently several scales to determine frailty in older people, within which the Fried frailty phenotype is positioned as a standard of physical frailty [18]. Other commonly used tools are the Rockwood Frailty Index, the Edmonton Frail Scale (EFS), and the Clinical Frailty Scale, which consists of simple questions and measurements for older people, such as weight, height, and walking speed, but the questions seem to impose greater complications since they are a more subjective measurement that will depend on the respondent’s self-appreciation and also, to a certain extent, influence how they feel emotionally at the time of the evaluation [19,20,21,22]. Likewise, there are various phenotypic determinants of the multidimensional construct of frailty (e.g., hepatic frailty, oral frailty, nutritional frailty, among others), that complicate its correct diagnosis [23,24,25]. In this context, different investigations indicate that the development of one or several biomarkers to detect frailty may be a more objective and precise way of evaluating both the initial state of the person and the progression of frailty over time, supporting the diagnosis, prognosis, and therapeutic decisions [26,27].

To date, different potential biomarkers of frailty have been reported, associated with the pathophysiological components of frailty, highlighting markers associated with increased inflammation, sarcopenia, nutritional deficit, and different hematological and biochemical parameters [28,29]. Based on the above, the main objective of this work was to describe the main biomarkers associated with frailty and to evaluate their possible usefulness for the early diagnosis of frailty in older people.

## 2. Methodology

We analyzed all original articles available in the mainly scientific databases (PubMed/Medline, Scopus, Web of Science, and SciELO), published until May 2022, with no date limitations and fulfilling the following inclusion criteria: (1) full text in English or Spanish; (2) original or review articles; (3) measurement of blood biomarkers; (4) frail patients (5) aging or older patients. When determining which articles to include, we analyzed the title and abstract and the full text of articles that fulfilled the inclusion criteria. The search terms employed were “frailty”, “biomarker” and “aging” or “older people”. Each article was evaluated by an independent reviewer, and any discrepancy was resolved by a second reviewer.

## 3. Diagnosis of Frailty: Main Diagnostic Tools and Their Deficiencies

Among all of the definitions of frailty, the frailty phenotype of Fried is the most used [30]. The Fried frailty phenotype evaluates five components to define frailty, which are: involuntary weight loss, weakness, reduction in walking speed, low physical activity, and fatigue [18]. This phenotype has recently been defined as the standard only for physical frailty, not considering intrinsic characteristics of frailty and being little applicable in the geriatric clinical area [31]. This is because this phenotype leaves out different areas affected by frailty, such as the nervous system and cognitive status, among others, in addition to not including psychosocial components [31,32]. On the other hand, the Frailty Index or Clinical Frailty Scale is based on a model of cumulative deficit that is defended by Mitnitski and Rockwood using the Canadian Study of Health and Aging [19]. Unlike the Fried frailty phenotype, this cumulative deficit approach describes frailty as a state that is caused by poor health throughout life, and the more deficits people have in their health, the more likely they are to develop frailty [30]. Despite its potential for clinical use, this tool presents a long list of deficit assessments, which is not very workable in geriatric practice, and its implementation in the health care area is not practical since it combines several domains [33,34]. The physical frailty phenotype and multidomain frailty index differ in that the physical frailty phenotype considers disability and comorbidities as a result of frailty, whereas in the multidimensional frailty model, these may be components of the model [35].

On the other hand, there is also the Edmonton Frail Scale (EFS), which was developed at the University of Alberta in the Canadian city of Edmonton by Rolfson and collaborators [20]. The EFS is a multidimensional frailty assessment tool designed to assist in the assessment and detection of frail older patients in primary health care [36]. This consists of 11 questions in 9 different domains, which are cognition, functional performance, general health status, functional independence, social support, pharmacological condition, nutritional aspect, mental condition, and continence [37,38]. Like the two previous tools, the EFS has deficiencies due to the subjectivity in some of its items and its limited usefulness in clinical implementation [14].

The presented evidence indicates that these scales, commonly used in research, are difficult to implement in clinical practice, for which it is necessary to have one or a set of biomarkers that can help predict the risk of frailty in time so that appropriate prevention or intervention measures can be implemented to reduce the risk or degree of frailty [39].

## 4. Blood Biomarkers Associated with Frailty

Age-associated changes in multiple physiological systems play a key role in the development of frailty, especially the neuromuscular, neuroendocrine, and immune systems, leading to compromise the physiological homeostasis [40,41]. The frailty phenotype includes sarcopenia, anorexia, osteoporosis, fatigue, risk of falling, poor physical health, and cognitive impairment, among other negative characteristics [40,41]. Just as frailty can be determined through different scales, the discovery of potential biomarkers of frailty is also important, which is evidenced in the multiple studies that analyzed inflammatory, hematological, and metabolic biomarkers, among others [42].

**Figure 1:**
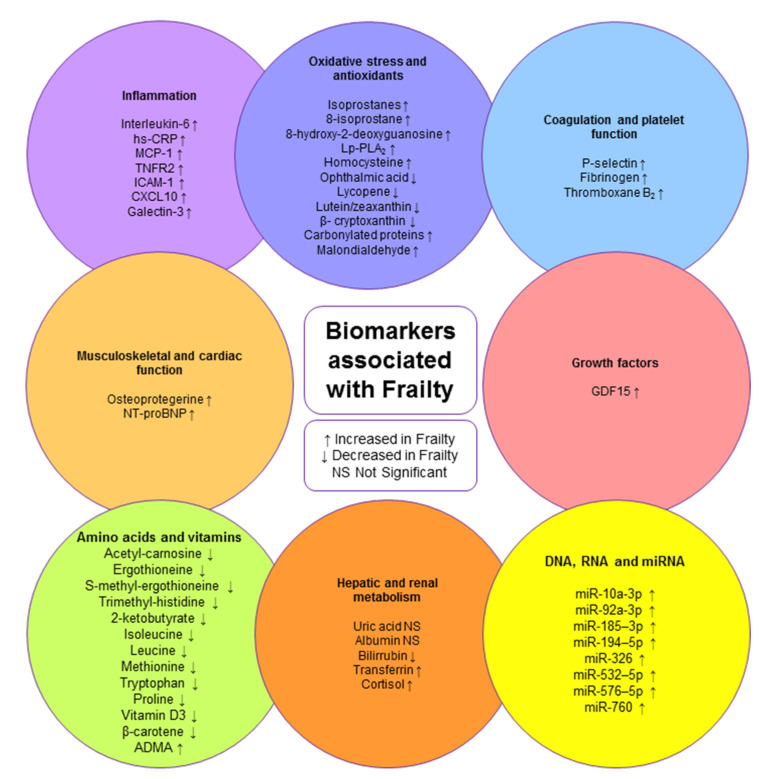
Biomarkers associated with frailty.

Next, the biomarkers associated with frailty and inflammation, antioxidants and oxidative stress, coagulation and platelet function, growth factors, skeletal and cardiac muscle function, amino acids and vitamins, hepatic and renal metabolism, and miRNA will be described (Figure 1).

### 4.1. Biomarkers Associated with Inflammation

Aging has been associated with changes in the immune system and inflammation level, where in the latter an increase in blood or serum concentrations of acute-phase proteins can be observed, such as C-reactive protein and pro-inflammatory cytokines [43,44,45,46]. Among the inflammation markers, it is possible to mention the pro-inflammatory cytokine interleukin-6 (IL-6), which contributes to host defense through stimulation of the acute-phase response, hematopoiesis, and immune reactions [47,48]. In a study evaluating the relationship between frailty and biomarkers of inflammation in older people undergoing surgical procedures (*n* = 137), high levels of IL-6 were found in frail patients; previous studies also established a relationship between slower walking speed and higher levels of IL-6, as well as reduced physical function [49,50]. In clinical practice, the high-sensitivity C-reactive protein (hs-CRP) biomarker is very useful for detecting vascular inflammation, and also low-grade inflammation [51,52]. A study of 1478 elderly people (70 to 84 years old) with the objective of evaluating the relationship between frailty and the biomarker of inflammation hs-CRP indicated that higher levels of hs-CRP were observed in frail older people (4.2 mg/L) [28]. The significant association of hs-CRP with frailty observed in this study is consistent with evidence of sustained systemic inflammation in frail patients [28,53,54,55].

In a study carried out in participants aged ≥60 years and classified into three groups, frail, *n* = 142; pre-frail, *n* = 864; and non-frail, *n* = 913, the relationship between frailty and inflammation was evaluated, determining the levels of three biomarkers: monocyte chemoattractant protein-1 (MCP-1), tumor necrosis factor receptor 2 (TNFR2), and intercellular adhesion molecule 1 (ICAM-1). The median results obtained for the MCP-1 biomarker were 415 pg/mL (frail), 384 pg/mL (pre-frail), and 364 pg/mL (non-frail), underlining an increase in the levels of MCP-1 in the frail group [50]. MCP-1 is known to be an important chemokine for macrophage recruitment and is produced by a variety of cell types, either constitutively or after induction by oxidative stress, cytokines, or growth factors [56,57]. In the same study, the results for TNFR2 (median) were 3151 pg/mL for frail individuals, 2669 pg/mL for pre-frail individuals, and 2385 pg/mL for non-frail individuals [50]. Tumor necrosis factor α (TNF-α) is a cytokine that participates in systemic inflammation, having two receptors, TNFR1 and TNFR2, and by binding to these, it can activate pro-apoptotic or anti-apoptotic signaling [58,59]. TNFR2 is a surface receptor that regulates cell survival and proliferation [60]. ICAM-1, a 90-kDa member of the immunoglobulin superfamily, was also analyzed in this group. It is essential for the firm arrest and transmigration of leukocytes out of blood vessels and into fabrics, is present in atherosclerotic lesions, and is involved in their progression [61]. The results (median) were 307 ng/mL for the frail, 293 ng/mL for the pre-frail, and 270 ng/mL for the non-frail; it was possible to observe that the frail elderly had higher values compared to the group of non-frail older people [50].

Other chemokines that are abundantly secreted by immune and non-immune cells in response to an inflammatory stimulus are IFN-γ-inducible protein 10 (CXCL-10), TNF-α, and interleukin 1 beta (IL-1β) [62,63]; these are involved in the pathogenesis of various inflammatory diseases and are also potent chemoattractants for Th1 lymphocytes and natural killer (NK) cells [64,65]. In a study conducted in a group of 32 participants (frail group, *n* = 16; non-frail group, *n* = 16) to assess the relationship between frailty and inflammation, the CXCL-10 biomarker was found to be increased in frailty, which suggests the possibility that the activation of inflammation in frailty is probably mediated by Th1 lymphocytes [66].

It is also possible to mention another marker of inflammation, galectin-3, a β-galactoside binding lectin widely expressed in immune, epithelial, and endothelial cells and sensory neurons that plays an important role in the exacerbation of autoimmune/inflammatory diseases, malignant cardiac fibrosis, and the progression of heart failure [67,68,69,70]. In a study carried out in a group of 54 frail and 74 non-frail persons, the relationship between frailty and inflammation was evaluated, and it was observed that galectin-3 was increased in the frail group, for which the authors suggested that galectin-3 upregulation was associated with the development of heart failure and could interfere with mechanisms of premature senescence, accelerated aging and development of frailty [71].

The descriptions of all of the above-mentioned biomarkers are presented in Table 1.

### 4.2. Biomarkers Associated with Oxidative Stress and Antioxidants

Free radicals are highly reactive atoms or molecules that have one or more unpaired electrons in their outer shell, while the terms reactive oxygen species (ROS) and nitrogen species (RNS) refer to radicals derived from oxygen and nitrogen [72,73].

Isoprostanes, osteoprotegerin, and lipoprotein-associated phospholipase A_2_ (Lp-PLA2), a well-described biomarker of increased oxidative stress, were evaluated in the Framingham Offspring Study (≥60 years, three groups classified by Fried frailty phenotype: frail, *n* = 142; pre-frail, *n* = 864; non-frail, *n* = 913), and the results show that increased levels of isoprostanes and Lp-PLA2 were associated with higher odds of frailty, while high levels of osteoprotegerin were associated with slower walking speed [50].

Isoprostanes are prostaglandin-like compounds formed in vivo by free radical-initiated non-enzymatic peroxidation of arachidonic acid [74]. For the Framingham study, the median results obtained were 11.5 mg/L for the frail, 10.2 mg/L for the pre-frail, and 9.5 mg/L for the non-frail, with higher values found in the frail elderly [50]. 8-Isoprostane is a marker of the eicosanoid family, produced non-enzymatically by successive oxidations in membrane phospholipids [75]. In a study that included 58 participants ≥65 years (*n* = 29 frail, *n* = 29 non-frail), high plasmatic levels of 8-isoprostane were observed in the frail group (frail group: 69.5 pg/mL, interquartile range: 64.0–96.7 pg/mL) (control group: 51.3 pg/mL, interquartile range: 38.2–72.2 pg/mL); however, it extensive studies are still necessary to help establish a better relationship between these [76].

On the other hand, it is also important to mention that 8-hydroxy-2-deoxyguanosine (8-OHdG), which is generated after the repair of DNA damage mediated by reactive oxygen species, is one of the biomarkers of the most widely recognized oxidative DNA damage [77]. A study conducted in Japan, which included a group of 140 outpatients with probable Alzheimer’s disease, showed evidence of significant differences in 8-OHdG between frailty states: frail, 5.39 ± 2.23; pre-frail, 5.44 ± 2.70; and non-frail, 3.90 ± 1.67 [78]. In addition, the authors pointed out that both 8-OHdG and 8-isoprostane levels were indicative of oxidative damage of DNA and lipids associated with the progression of frailty in patients with Alzheimer’s disease [78].

Lp-PLA2 is a member enzyme of the 50 kDa phospholipase A₂ family that hydrolyzes oxidized phospholipids and is mainly associated with low-density lipoprotein (LDL), participating in oxidative modification of the vascular wall that can promote vascular inflammation and development of atherosclerotic plaque [79,80,81]. In the Framingham study described above, the median results obtained for Lp-PLA2 were 210 ng/mL (Frail),199 ng/mL (Pre-frail), and 199 ng/mL (Non-frail), with higher values in frail older people, which was also associated with a slower walking speed [50]. This relationship could be explained by previous studies, where murine models have shown that changes in Lp-PLA2 levels affect muscle contractility and resistance [82].

In a study carried out in a group of 1211 elderly people from the Toledo Study of Healthy Aging, the authors evaluated the relationship between frailty and oxidative stress, and in the group of frail people, they found a positive correlation between homocysteine levels, frailty, and the components of the fatigue score [83]. These findings are consistent with another study suggesting that inflammation could be the key mechanism in the pathophysiology of frailty [84]. Regarding homocysteine, high levels of this biomarker are related to inflammation and vitamin B deficiency, oxidative stress, mitochondrial dysfunction, and altered DNA methylation [85]. The above factors are also associated with cardiovascular diseases, disability, and a high risk of death [83].

In a study conducted to evaluate the relationship between frailty and oxidative stress in a group of 19 participants, of which 9 were diagnosed as frail and 10 as non-frail according to the Edmonton Frail Scale (EFS), a significant decrease in the plasmatic ophthalmic acid level was observed in the group of frail older people [86]. An important point about this biomarker is that it is one of the most abundant antioxidants in cells [87]. Ophthalmic acid (l-γ-glutamyl-l-α-amino butyryl glycine) is an analog of glutathione in which cysteine is replaced by l-α-aminobutyrate; since it is released from the liver into the bloodstream, its increase in blood has been proposed as an indicator of hepatic glutathione depletion [88].

The redox balance is altered in frail elderly people, showing that natural antioxidants that can be incorporated into the diet, such as lycopene, lutein/zeaxanthin, and β-cryptoxanthin, can be affected. This fact was observed in the FRALOMIC study, which was composed of 1450 people over 65 years of age [39]. Lycopene is a phytochemical found mainly in tomatoes and has been associated with anticancer, antidiabetic, antioxidant, cardioprotective, anti-inflammatory, hepatoprotective, neuroprotective, and other biological effects [89,90]. Lutein/zeaxanthin are two fat-soluble antioxidants that are the main components of macular pigment, a compound concentrated in the macular region of the retina that is responsible for the vision of fine features [91]. β-cryptoxanthin, a carotenoid commonly found in fruits, blood, and tissues, is an antioxidant and protects organs and tissues from oxidative damage; however, its main function is as a precursor of vitamin A [92,93]. The levels of lycopene (non-frail: 0.40 µmol/L, pre-frail: 0.36 µmol/L, frail: 0.29 µmol/L), lutein/zeaxanthin (non-frail: 0.36 µmol/L, pre-frail: 0.33 µmol/L, frail: 0.27 µmol/L) and β-cryptoxanthin (non-frail: 0.22 µmol/L, pre-frail: 0.21 µmol/L, frail: 0.16 µmol/L) were found to be significant decreased in the frail group, where the β-cryptoxanthin level was the lowest [29]. In this sense, the damage caused by oxidative stress represents a possible trigger for the genesis of the frailty syndrome, which can also be supported by the low levels of lycopene, lutein/zeaxanthin, and β-cryptoxanthin in plasm of frail patients. Continuing with the study of the FRAILOMIC group, the association between frailty and the marker carbonylated proteins in older people was studied [94]. This biomarker represents an irreversible form of protein modification, is relatively stable, and is called a biomarker of protein oxidation [95]. High levels of carbonylated proteins were observed in the frail group, compared to the pre-frail and non-frail groups (non-frail group: 0.26 nmol/mg, pre-frail group: 0.30 nmol/mg, frail group: 0.31 nmol/mg) [29]. With the data obtained, the researchers concluded that low concentrations of antioxidants and high concentrations of carbonylated proteins were associated with pre-frail and frail people over 65 years of age, thus suggesting that a diet high in these antioxidants could support frailty prevention [29].

Lipids are very sensitive to attack by oxidants, and to date, malondialdehyde (MDA), 4-hydroxy-2-nonenal, and F2-isoprostane have been the main biomarkers used for the evaluation of lipid peroxidation [96]. In the Toledo Study for Healthy Aging, in which 742 people were evaluated to assess the relationship between frailty and oxidative stress markers, increased levels of MDA were found in the frail group [97]. MDA is a final product of lipid peroxidation and is considered a biological marker of oxidative stress [98,99]. Therefore, according to a previous study, MDA is the marker that best shows the evolution of damage caused by lipid peroxidation, showing an exponential increase with age, which is associated with high systemic oxidative stress [100].

Based on the above, oxidative stress could contribute to a decline in muscle mass and function with the aging process, leading to weakness and slowness [101]. The descriptions of all of the above-mentioned biomarkers are presented in Table 2.

### 4.3. Biomarkers Associated with Coagulation and Platelet Function

Hemostasis stops bleeding at the site of vascular injury and maintains blood vessel integrity through clot formation, where this physiological process consists of interactions between endothelial cells, platelets, von Willebrand factor, and coagulation factors [102]. Some markers indicate when the platelet is activated, among which we can mention P-selectin (CD62P); some proteins participate in coagulation, such as fibrinogen and eicosanoids released by the activated platelet, such as thromboxane A_2_ [103,104,105].

In a study conducted on a group of people over 60 years of age, the cohort was divided into 3 groups: frail, *n* = 142; pre-frail, *n* = 864; non-frail, *n* = 913. This research aimed to evaluate the relationship between frailty and platelet function, observing that the median results for P-selectin in the group of frail and pre-frail people were greater than that in the non-frail group [50]. P-selectin belongs to the selectin family (CD62) and is a protein stored in the α-granules of platelets and the Weibel Palade bodies of endothelial cells [106].

A study carried out on elderly patients (≥65 years) from the retirement community of San Antonio (TX, USA) evaluated the relationship between frailty and coagulation, observing higher fibrinogen levels in the frail group [107]. Fibrinogen is a glycoprotein, synthesized in the liver, whose main function is to stop excessive bleeding and is associated with the thrombosis process [108,109]. In another study, where the relationship between frailty and platelet activation was evaluated (frail, *n* = 29; non-frail, *n* = 29), it was observed that the frail group had higher plasma levels of thromboxane B_2_ (TXB2) (69.5 pg/mL) compared to the non-frail group (51.3 pg/mL) [76]. Thromboxane B_2_ is a non-enzymatic derivative of thromboxane A_2_ (TXA2), the latter produced from arachidonic acid [76].

The previous results show that in the group of frail older people, the levels of P-selectin, fibrinogen, and thromboxane B_2_ were higher than in the group of non-frail older people, which is important to consider since frailty can cause the platelets in these people to be more activated with a higher risk of thrombosis, there being a clear relationship between platelet hyper-reactivity and the occurrence of thromboembolic events, and can contribute to cardiovascular comorbidities in old age [110,111]. Frailty is closely related to oxidative stress, which plays an important role in the mechanism of platelet activation; therefore, some researchers point out that significant changes in oxidative stress may be one of the main causes of a high platelet response in frail patients [112,113]. Descriptions of all of the above-mentioned biomarkers are presented in Table 3.

### 4.4. Biomarkers Associated with Growth Factors

A study of 58 participants (age 65 years or more) (frail group, *n* = 29; non-frail group, *n* = 29) evaluated the relationship between frailty and growth differentiation factor 15 (GDF-15) [76]. The evidence showed that the frail group presented higher levels in plasma (median: 2379 pg/mL) than the non-frail group (median: 1367 pg/mL) [76]. GDF15 is a member of the transforming growth factor-beta superfamily, widely distributed in mammalian tissues, and has been shown to play multiple roles in various pathologies, including inflammation, cancer, cardiovascular diseases, and obesity [114]. Being a stress-inducible cytokine, GDF-15 is upregulated by several proteins associated with stress and inflammation, such as IL-1β, TNF-α, IL-2, and macrophage colony-stimulating factor (MCSF-1), suggesting a complex tissue-specific regulation [115]. GDF-15 levels increase with age, which is why it has been proposed as a potential biomarker of aging and mitochondrial dysfunction [76,116]. In a previous study that evaluated older patients with chronic kidney disease, it was observed that there was a correlation between age and GDF-15 levels, which could serve as a precedent to evaluate the relationship between high levels of GDF-15 and frailty in extensive cohorts [117]. The description of this study is shown in Table 4.

### 4.5. Biomarkers Associated with Musculoskeletal and Cardiac Function

The Framingham study described above also was to assess the relationship between frailty and musculoskeletal function, and the median results obtained for biomarker osteoprotegerin were 5.88 pm/L for the frail group, 5.13 pm/L for the pre-frail group, and 4.81 pm/L for the non-frail group, showing that it was higher in the frail group [50]. Concerning this, some authors report that acute secondary events due to fracture had an additive and independent effect by accentuating the differences between frailty groups, where the admitted patients with fractures had higher mean values of osteoprotegerin than patients without fractures [118]. Osteoprotegerin is a glycoprotein involved in bone remodeling that acts as a decoy receptor for receptor activator for nuclear factor κ B Ligand (RANKL), inhibiting bone resorption [119,120]. As mentioned, osteoprotegerin has been described as a biomarker of bone remodeling, but there is also evidence that it plays a role in cardiovascular diseases, contributing to atherosclerosis and being associated with peripheral arterial disease, where the latter has been associated with slower walking speeds [121,122,123].

NT-proBNP is an N-terminal pro-brain natriuretic peptide that is secreted by cardiac myocytes in response to ventricular and atrial wall stress, serving as a serum biomarker for the diagnosis of heart failure [124]. In a study carried out in a group of 54 frail participants and 74 non-frail participants, the relationship between frailty and cardiac function was evaluated, and the authors observed that NT-proBNP level was increased in the frail group [71]. In the same study, NT-proBNP emerged as an independent marker associated with frailty and has also been suggested as a useful biomarker in identifying frailty in patients with multiple myeloma [71,125]. In another study, it was observed that frail older people had more comorbidities, lower body mass index (BMI), and lower hemoglobin, and also had elevated levels of NT-proBNP along with more severe symptoms of chronic heart failure [126]. Descriptions of all of the above-mentioned biomarkers are presented in Table 5.

### 4.6. Biomarkers Associated with Amino Acids and Vitamins

An adequate protein diet is important in the elderly to maintain muscle mass, as well as support wound healing, skin integrity, immunity, and recovery from illness [127]. In addition, older people also need high protein intake to be able to compensate for high metabolism in conditions of inflammation and the decrease in anabolic capacity with aging [128]; in addition, decreased protein intake has been associated with sarcopenia and frailty [129,130]. Moreover, protein intake influences the production of IGF-1, an important trophic hormone with growth-promoting effects in almost all cells of the body, especially skeletal muscle, cartilage, and bone.

A study carried out in a group of 19 participants, of which 9 were diagnosed as frail and 10 as non-frail according to the Edmonton Frail Scale (EFS), assessed the relationship between frailty and the following amino acids: carnosine, ergothioneine, s-methyl-ergothioneine, trimethyl-histidine, 2-ketobutyrate, isoleucine, leucine, methionine, tryptophan, and proline; all of the aforementioned analytes were decreased in the frail group [86].

Relative to acetyl-carnosine, N-acetyl derivatives of histidine, such as carnosine, exist in mammalian cardiac and skeletal muscle, and the total concentration of these imidazoles can be within the measured range of L-carnosine in skeletal muscle (~10 mM) [131,132]. On the other hand, ergothioneine (ERG) is an unusual betaine thiohistidine amino acid that has potent antioxidant activities and also promotes neuronal stem cell differentiation [133,134,135,136]. As for trimethyl-histidine, it is formed by post-translational methylation of histidine residues in actin and myosin, and its plasma concentration and urinary excretion are sensitive markers of myofibrillar protein degradation [137,138]. On the other hand, the catabolism of threonine and methionine converges in 2-ketobutyrate, which was associated with cognitive impairment and low mobility, which are also associated with frailty in older people [139,140]. The amino acids isoleucine and leucine are branched-chain amino acids, which, together with methionine and tryptophan, are essential amino acids that cannot be synthesized in the human body, so they must be ingested in the diet [141,142,143,144,145,146]. Regarding these amino acids, leucine plays an important role in the regulation of protein metabolism [147], activating protein synthesis in the skeletal muscle by increasing phosphorylation of mammalian target of rapamycin (mTOR) [148], which is important to maintain muscle mass.

Finally, proline is necessary for the biosynthesis of collagen and other proline-containing proteins, so regulation of proline availability for collagen biosynthesis is essential to maintain tissue integrity [149]. The quality of the diet is inversely associated with the risk of developing frailty, and as it is a potentially modifiable factor, it can play an important role in frailty prevention [150,151,152].

A study conducted in the FRAILOMIC cohort evaluated the relationship between frailty and the levels of vitamin D and β-carotene [29]. Vitamin D exists in two forms: vitamin D2, which is obtained from UV radiation on the sterol of ergosterol-type yeasts and is found naturally in fungi exposed to the sun, while the influence of UVB light on the skin of humans synthesizes vitamin D3 [153]. Vitamin D acts as a steroid hormone and influences the bones, intestines, immune and cardiovascular systems, pancreas, muscles, brain, and the control of cell cycles [153]. β-carotene is a tetraterpenoid that in its structure includes 2 β-ionone rings; it is the most important source of non-preformed vitamin A [89]. Regarding these markers, both vitamin D and β-carotene levels were decreased in the frail group [29].

Finally, in a study carried out in a population of 1287 participants (frail group, *n* = 107; pre-frail group, *n* = 542; non-frail group, *n* = 638), the relationship between frailty and amino acid levels was evaluated, observing higher values for asymmetric dimethylarginine (ADMA) in the frail group [154]. ADMA is synthesized when arginine residues in proteins are methylated by the action of arginine methyltransferase, acting as an inhibitor of nitric oxide (NO) synthesis, reducing NO production, and this could lead to endothelial dysfunction and cardiovascular events [155,156,157]. As mentioned above, ADMA influences the reduction of NO production, leading to endothelial dysfunction and cardiovascular events. Descriptions of all of the above-mentioned biomarkers are presented in Table 6.

### 4.7. Biomarkers Associated with Hepatic and Renal Metabolism

A study carried out on a group of 140 patients with probable Alzheimer’s disease (age ≥65 years) evaluated the relationship between frailty and hepatic metabolism; patients underwent uric acid, albumin, and bilirubin analysis [78]. For uric acid and albumin biomarkers, no significant differences were found between the frail and other groups [78]. Decreased albumin values in clinical settings are associated with nutritional deterioration and inflammatory response associated with the disease, which could be the result of the aging process; however, more studies are still needed to establish this correlation with frailty [158].

On the other hand, in this same study, significant differences were found for bilirubin, with a lower level in the frail group (0.54 ± 0.18 mg/dL), compared to the pre-frail group (0.60 ± 0.21 mg/dL) and not frail (0.66 ± 0.22 mg/dL) [78]. In another study conducted on a group of 19 participants, where 9 were diagnosed as frail (age 88.2 ± 6.8 years) and 10 as non-frail (age 80.5 ± 4.7 years) according to the Edmonton Frail Scale (EFS), it was observed that the bilirubin level was decreased in the frail group, confirming the findings described in the above study [86]. Bilirubin is an end product of the catabolism of the heme group of hemoglobin present in senescent red blood cells [159]. The presented evidence suggests that low serum bilirubin levels could decrease antioxidant capacity, leading to several diseases associated with oxidative stress, and increased levels of oxidative stress and pro-inflammatory conditions in frail and pre-frail patients [112,160].

On the other hand, a previously mentioned study of elderly patients from the retirement community in San Antonio (Texas) also evaluated the relationship between frailty and liver metabolism, observing higher levels of transferrin (ng/mL) in the frail group [107]. Transferrin is a 76 kDa glycoprotein, produced mainly in the liver, and its function corresponds to the transport of iron. [161].

Finally, a study carried out in a group of 252 elderly people from Spain evaluated the relationship between frailty and renal metabolism, and the authors observed that the frail group present higher levels of cortisol than the pre-frail and non-frail groups, which could correlate with the decreased ability to cope with the stress that occurs in frail patients [162]. Cortisol is one of the major glucocorticoids synthesized in the fasciculate zone of the adrenal cortex; it plays an important role in regulating the body’s response to stress through the maintenance of homeostasis and regulation of water and salt balance, blood pressure, immune function and metabolism [163,164]. Descriptions of all of the above-mentioned biomarkers are presented in Table 7.

### 4.8. Biomarkers Associated with DNA, RNA, and miRNA

MicroRNAs (miRNAs) are a class of non-coding RNAs with a length of 22 nucleotides; they influence the expressions of their target mRNAs at post-transcriptional levels and have been used to detect genetic biomarkers of healthy aging and longevity [165,166]. On the other hand, it is also possible to find miRNA derived from exosomes in extracellular fluids that originate when the multivesicular bodies fuse with the plasma membrane [167]. These exosomes contain miRNA from the cells that gave rise to them, which can modulate protein production in recipient cells [168]. In a study of young people (23–35 years) and older people (≥65 years) classified as frail or robust according to Fried’s criteria [167], miRNAs derived from exosomes were analyzed, and the results show that eight miRNAs were uniquely enriched in frail patients: miR-10a-3p, miR-92a-3p, miR-185–3p, miR-194–5p, miR-326, miR-532–5p, miR-576–5p and miR-760 [167]. Regarding miR-10a-3p, it has been reported that it can regulate cholesterol metabolism in chondrocytes, while miR-92a-3p is expressed during chondrogenesis in human mesenchymal stem cells and differentially in osteoarthritis [169,170]. miR-185-3p has been identified as a tumor suppressor in colorectal cancer [171], whereas miR-194 is a specific miRNA in vertebrates that has a fundamental role in energy production, inflammatory inhibition, and neoplasms such as breast cancer, non-small cell lung cancer, and colorectal cancer [172,173,174]. On the other hand, miR-194-5p has been identified as a regulator of tumorigenesis, while recent studies have suggested that the downregulation of miR-326 serves as a tumor suppressor mechanism and plays a crucial role in carcinogenic cell growth [175,176]. miR-532-5p was reported to have a cancer-promoting effect in cutaneous melanoma [177]; while miR-576-5p enhances melanoma cell invasion in vitro, it is also upregulated in esophageal cancer, where it was found to be involved in cancer cell migration and invasion [178,179]. miR-760 has been reported to regulate the proliferation and apoptosis of a variety of cells, including pulmonary artery smooth muscle cells and colon and ovarian cancer cells [180].

All previously mentioned miRNAs were observed to be augmented in the frail group, representing novel biomarker candidates for frailty [167]. More research is required to determine if these miRNAs change before the development of frailty, which could help determine whether they should be considered as biomarkers for the early diagnosis of frailty. Descriptions of these biomarkers are provided in Table 8.

## 5. Outlook and Future Directions

Frailty has been assessed from a biophysical frailty phenotype approach as well as a cumulative deficit index model, both of which are reproducible and can predict important health outcomes, such as mortality, chronic diseases, and functional dependency [181,182]. Several biomarkers have been studied as possible candidates to determine frailty in older people; however, using them individually can be a limitation because these biomarkers are mostly considered markers of aging, for which alterations could be associated with this process regardless of whether or not they involve frailty [27,183]. Although many of the biomarkers mentioned are associated with frailty, none of them alone is sufficient for its diagnosis, but together they could improve the sensitivity and specificity of frailty detection, which is also consistent with the definition of frailty as a multidimensional syndrome caused by alterations in multiple biological systems [184]. This has been similarly observed in the relationship between diabetes mellitus and salivary biomarkers, when the incorporation of salivary amylase quantification could reflect improving glycemic control [185]. Communication and information technologies have been proposed in recent years as potential interventions to support older people who are affected by various clinical conditions that can impact their well-being [186]. The application of artificial intelligence (AI) technology offers a potential solution to improve the identification of frailty [187]. AI can be defined as a branch of computer science whose objective is to create systems or methods that analyze information and allow the management of complexity in a wide range of applications [188]. Previous studies suggest that the use of a predictive model based on machine learning could be useful to detect future frailty conditions, as well as the risk of hospital readmissions of these patients, using both clinical and socioeconomic variables that can generally be collected in centers for health care [189,190].

In the clinical environment, there is no standard measure of frailty, and it is necessary to have integral diagnostic tools that allow frailty to be predicted with consideration for the characteristics of each population and that can also be used by employing patient records that health centers maintain [190].

For this reason, there is a need for more research that will allow us to have validated biomarkers of frailty, which, together with some of the frailty scales currently available, can be used in all health centers to detect the risk of frailty, and in this way prevent this syndrome and its associated adverse health events.

## 6. Conclusions

There are several scales to diagnose frailty, among which the most used is the Fried phenotype, but there is still no validated biomarker that contributes to this diagnosis. Several studies have been carried out to evaluate biomarkers that may be altered when an elderly person is already frail or maintained robustly, but there are still no specific biomarkers that can help in the early diagnosis of frailty. The development of frailty in older people leads to a loss of self-worth and, therefore, they become dependent on the care provided by other persons. In addition, the risk of falls is also increased, and the development of other diseases can lead to the elderly person having to be hospitalized, with all the risks that this implies, which also decreases their life expectancy and quality of life. Being able to determine a biomarker that can detect frailty early would greatly contribute to improving the quality of life of the elderly and thus reduce the incidence of frailty, which is why it is important to continue with more studies that support the recent evidence.

The evidence presented in this review demonstrates that biomarkers associated with inflammation, oxidative stress, skeletal/cardiac muscle function, and platelet function represent promising markers of frailty due to their pathophysiological association with this syndrome. To a lesser extent but with the possibility of greater innovation, biomarkers associated with growth factors, vitamins, amino acids, and miRNAs represent alternatives as markers of this geriatric syndrome. Likewise, the use of AI and the incorporation of sociodemographic variables associated with frailty and available in the records of health centers represent interesting methodologies to strengthen the diagnosis of frailty through the aforementioned biomarkers.

## Figures and Tables

**Table 1:** Biomarkers associated with inflammation.

BIOMARKER	SAMPLE	CUT VALUES (FRAIL—NOT FRAIL)	FRAILTY SCALE	COUNTRY	YEAR	GROUP DESCRIPTION	REFERENCE
Interleukin-6 (pg/L)	Serum	Median 11.08 interquartile range: 5.04–13.63 (Frail) Median 4.11 interquartile range: 2.83–5.84 (Not Frail)	Fried	Norway	2010	Transversal study Group of 137 patients, median age 80 years	Rønning, B. et al., 2010 [49]
Hs-CRP (mg/L)	Serum	4.2 (Frail) 3.1 (Not Frail)	Fried	China	2016	Transversal study Group of 1478 participants, 70–84 years old	Zhu, Y. et al. (2016) [28]
MCP-1 (pg/mL)	Plasma	Median and interquartile range: 415 (345–501) (Frail) 384 (324–462) (Pre-frail) 364 (301–441) (Not Frail)	Fried	United States	2016	Group of people over 60 years of age, divided into 3 groups: *n* = 142 frail, *n* = 864 pre-frail, *n* = 913 not frail	Liu, C.K. et al. (2016) [50]
TNFR2 (pg/mL)	Plasma	Median and interquartile range: 3151 (2506–4447) (Frail) 2669 (2149–3443) (Pre-frail) 2385 (1963–2877) (Not Frail)	Fried	United States	2016	Group of people over 60 years of age, divided into 3 groups: *n* = 142 frail, *n* = 864 pre-frail, *n* = 913 not frail	Liu, C.K. et al. (2016) [50]
ICAM-1 (ng/mL)	Plasma	Median and interquartile range: 307 (250–381) (Frail) 293 (244–359) (Pre-frail) 270 (233–334) (Not Frail)	Fried	United States	2016	Group of people over 60 years of age, divided into 3 groups: *n* = 142 frail, *n* = 864 pre-frail, *n* = 913 not frail	Liu, C.K. et al. (2016) [50]
CXCL10	Whole blood	Increased in frailty	Fried	United States	2009	Group of 32 people, 16 frail and 16 not frail, average age of 83 years, 87.5% women	Qu et al. (2009) [66]
Galectin-3 (ng/mL)	Serum	34.4 ± 19.3 (Frail). 14.3 ± 7.6 (Not Frail). Increased in frailty	Clinical Frailty Scale (CFS)	Italy	2020	Group of 54 frail persons and 74 not frail persons, age frail 70.5 ± 5.4 years, age not frail 68.2 ± 4.2 years	Komici et al. (2020) [71]

**Table 2:** Biomarkers associated with oxidative stress and antioxidants.

BIOMARKER	SAMPLE	CUT VALUES (FRAIL–NOT FRAIL)	FRAILTY SCALE	COUNTRY	YEAR	GROUP DESCRIPTION	REFERENCE
Isoprostanes (mg/L)	Urine	Median and interquartile range: 11.5 (8.50–15.40) (Frail) 10.2 (7.60–14.30) (Pre-frail) 9.5 (7.1–12.8) (Not Frail)	Fried	United States	2016	Group of people over 60 years of age, divided into 3 groups: *n* = 142 frail, *n* = 864 pre-frail, *n* = 913 not frail	Liu, C.K. et al. (2016) [50]
8-isoprostane (pg/mL)	Plasma	Interquartile range: 64.0–96.7 (Frail) 38.2–72.2 (Not Frail)	Fried	Chile	2019	Group of people over 65 years of age, divided into 2 groups: *n* = 29 frail, *n* = 29 not frail.	Arauna, D. et al. (2019) [76]
8-hydroxy-2-deoxyguanosine (ng/mg Creatinine)	Urine	5.39 ± 2.23 (Frail) 5.44 ± 2.70 (Pre-frail) 3.90 ± 1.67 (Not Frail)	Fried	Japan	2017	Group of 140 outpatients with probable Alzheimer’s disease, 65 years and older	Namioka, N. et al. (2017) [78]
Lp-PLA2 mass (ng/mL)	Plasma	Median and interquartile range: 210 (183–237) (Frail) 199 (172–229) (Pre-frail) 199 (168–228) (Not Frail)	Fried	United States	2016	Group of people over 60 years of age, divided into 3 groups: *n* = 142 frail, *n* = 864 pre-frail, *n* = 913 not frail	Liu, C.K. et al. (2016) [50]
Lp-PLA2 activity (nm/mL/min)	Plasma	Median and interquartile range: 139 (119–166) (Frail) 137 (115–160) (Pre-frail) 136 (114–159) (Not Frail)	Fried	United States	2016	Group of people over 60 years of age, divided into 3 groups: *n* = 142 frail, *n* = 864 pre-frail, *n* = 913 not frail	Liu, C.K. et al. (2016) [50]
Homocysteine (µmol/L)	Serum	Increased in frail older people	Fried	Spain	2020	Study group of 1211 people, of which 515 were men (42.5%) and 696 were women (57.5%), whose ages were between 65 and 98 years	Alvarez-Sanchez *n*, (2020) [83]
Ophthalmic acid	Serum	Decreased in frailty	Edmonton Frail Scale (EFS)	Japan	2020	Group of 19 participants, mean age 84.2 ± 6.9 years, 7 men and 12 women, of which 9 were diagnosed as frail (age 88.2 ± 6.8 years) and 10 as not frail (age 80.5 ± 4.7 years)	Kameda, M., et al. (2020) [86]
Lycopene (µmol/L)	Plasma	Decreased in frail older people	Fried	Germany	2019	Study group of 1450 people from the FRAILOMIC database, all over 65 years of age	Kochlik, B., (2019) [29]
Lutein/zeaxanthin (µmol/L)	Plasma	Decreased in frail older people	Fried	Germany	2019	Study group of 1450 people from the FRAILOMIC database, all over 65 years of age	Kochlik, B., (2019) [29]
β- cryptoxanthin (µmol/L)	Plasma	Decreased in frail older people	Fried	Germany	2019	Study group of 1450 people from the FRAILOMIC database, all over 65 years of age	Kochlik, B., (2019) [29]
Carbonylated proteins (nmol/mg)	Plasma	Increased in frail older people	Fried	Germany	2019	Study group of 1450 people from the FRAILOMIC database, all over 65 years of age	Kochlik, B., (2019) [29]
Malondialdehyde	Plasma	Increased in frail older people	Fried	Spain	2014	Group of 742 people from the Toledo Study for Healthy Aging, of which 309 were men and 433 were women	Ingles, M., (2014) [97]

**Table 3:** Biomarkers associated with coagulation and platelet function.

BIOMARKER	SAMPLE	CUT VALUES (FRAIL—NOT FRAIL)	FRAILTY SCALE	COUNTRY	YEAR	GROUP DESCRIPTION	REFERENCE
P-selectin (ng/mL)	Plasma	Median and interquartile range: 41 (32–51) (Frail) 41 (33–49) (Pre-frail) 39 (32–47) (Not Frail)	Fried	United States	2016	Group of people over 60 years of age, divided into 3 groups: *n* = 142 frail, *n* = 864 pre-frail, *n* = 913 not frail	Liu, C.K. et al. (2016) [50]
Fibrinogen (g/L)	Plasma	70.4 ± 17.5 (Frail) 40.6 ± 9.3 (Not Frail)	Fried	United States	2013	Group of 65 people over 65 years old *n* = 12 frail *n* = 22 not frail	Darvin, K. et al., 2013 [107]
Thromboxane B_2_ (ng/mL)	Plasma	Interquartile range: 64.0–96.7 (Frail) 38.1–72.2 (Not Frail)	Fried	Chile	2019	Group of people over 65 years of age, divided into 2 groups: *n* = 29 frail, *n* = 29 not frail	Arauna, D. et al. (2019) [76]

**Table 4:** Biomarkers associated with growth factors.

BIOMARKER	SAMPLE	CUT VALUES (FRAIL—NOT FRAIL)	FRAILTY SCALE	COUNTRY	YEAR	GROUP DESCRIPTION	REFERENCE
GDF15	Plasma	Interquartile range: 1845–4121 (Frail) 1190–1747 (Not Frail)	Fried	Chile	2019	Group of people over 65 years of age, divided into 2 groups: *n* = 29 frail, *n* = 29 not frail	Arauna, D. et al. (2019) [76]

**Table 5:** Biomarkers associated with musculoskeletal and cardiac function.

BIOMARKER	SAMPLE	CUT VALUES (FRAIL—NOT FRAIL)	FRAILTY SCALE	COUNTRY	YEAR	GROUP DESCRIPTION	REFERENCE
Osteoprotegerine (pm/L)	Plasma	Median and interquartile range: 5.88 (4.82–7.41) (Frail) 5.13 (4.23–6.13) (Pre-frail) 4.81 (4.01–5.59) (Not Frail)	Fried	United States	2016	Group of people over 60 years of age, divided into 3 groups: *n* = 142 frail, *n* = 864 pre-frail, *n* = 913 not frail	Liu, C.K. et al. (2016) [50]
NT-proBNP (pg/mL)	Serum	11,427.9 ± 21,803.4 (Frail). 1856.4 ± 3570.1 (Not Frail). Increased in frailty	Clinical Frailty Scale (CFS)	Italy	2020	Group of 54 frail persons and 74 not frail persons, age frail 70.5 ± 5.4 years, age not frail 68.2 ± 4.2 years	Komici et al. (2020) [71]

**Table 6:** Biomarkers associated with amino acids and vitamins.

BIOMARKER	SAMPLE	CUT VALUES (FRAIL—NOT FRAIL)	FRAILTY SCALE	COUNTRY	YEAR	GROUP DESCRIPTION	REFERENCE
Acetyl-carnosine	Serum	Decreased in frailty	Edmonton Frail Scale (EFS)	Japan	2020	Group of 19 participants, mean age 84.2 ± 6.9 years, 7 men and 12 women, of which 9 were diagnosed as frail (age 88.2 ± 6.8 years) and 10 as not frail (age 80.5 ± 4.7 years)	Kameda, M., et al. (2020) [86]
Ergothioneine	Serum	Decreased in frailty	Edmonton Frail Scale (EFS)	Japan	2020	Group of 19 participants, mean age 84.2 ± 6.9 years, 7 men and 12 women, of which 9 were diagnosed as frail (age 88.2 ± 6.8 years) and 10 as not frail (age 80.5 ± 4.7 years)	Kameda, M., et al. (2020) [86]
S-methyl-ergothioneine	Serum	Decreased in frailty	Edmonton Frail Scale (EFS)	Japan	2020	Group of 19 participants, mean age 84.2 ± 6.9 years, 7 men and 12 women, of which 9 were diagnosed as frail (age 88.2 ± 6.8 years) and 10 as not frail (age 80.5 ± 4.7 years)	Kameda, M., et al. (2020) [86]
Trimethyl-histidine	Serum	Decreased in frailty	Edmonton Frail Scale (EFS)	Japan	2020	Group of 19 participants, mean age 84.2 ± 6.9 years, 7 men and 12 women, of which 9 were diagnosed as frail (age 88.2 ± 6.8 years) and 10 as not frail (age 80.5 ± 4.7 years)	Kameda, M., et al. (2020) [86]
2-ketobutyrate	Serum	Decreased in frailty	Edmonton Frail Scale (EFS)	Japan	2020	Group of 19 participants, mean age 84.2 ± 6.9 years, 7 men and 12 women, of which 9 were diagnosed as frail (age 88.2 ± 6.8 years) and 10 as not frail (age 80.5 ± 4.7 years)	Kameda, M., et al. (2020) [86]
Isoleucine	Serum	Decreased in frailty	Edmonton Frail Scale (EFS)	Japan	2020	Group of 19 participants, mean age 84.2 ± 6.9 years, 7 men and 12 women, of which 9 were diagnosed as frail (age 88.2 ± 6.8 years) and 10 as not frail (age 80.5 ± 4.7 years)	Kameda, M., et al. (2020) [86]
Leucine	Serum	Decreased in frailty	Edmonton Frail Scale (EFS)	Japan	2020	Group of 19 participants, mean age 84.2 ± 6.9 years, 7 men and 12 women, of which 9 were diagnosed as frail (age 88.2 ± 6.8 years) and 10 as not frail (age 80.5 ± 4.7 years)	Kameda, M., et al. (2020) [86]
Methionine	Serum	Decreased in frailty	Edmonton Frail Scale (EFS)	Japan	2020	Group of 19 participants, mean age 84.2 ± 6.9 years, 7 men and 12 women, of which 9 were diagnosed as frail (age 88.2 ± 6.8 years) and 10 as not frail (age 80.5 ± 4.7 years)	Kameda, M., et al. (2020) [86]
Tryptophan	Serum	Decreased in frailty	Edmonton Frail Scale (EFS)	Japan	2020	Group of 19 participants, mean age 84.2 ± 6.9 years, 7 men and 12 women, of which 9 were diagnosed as frail (age 88.2 ± 6.8 years) and 10 as not frail (age 80.5 ± 4.7 years)	Kameda, M., et al. (2020) [86]
Proline	Serum	Decreased in frailty	Edmonton Frail Scale (EFS)	Japan	2020	Group of 19 participants, mean age 84.2 ± 6.9 years, 7 men and 12 women, of which 9 were diagnosed as frail (age 88.2 ± 6.8 years) and 10 as not frail (age 80.5 ± 4.7 years)	Kameda, M., et al. (2020) [86]
Vitamin D3 (nmol/L)	Plasma	Decreased in frail older people	Fried	Germany	2019	Study group of 1450 people from the FRAILOMIC database, all over 65 years of age	Kochlik, B., (2019) [29]
β-carotene (µmol/L)	Plasma	Decreased in frail older people	Fried	Germany	2019	Study group of 1450 people from the FRAILOMIC database, all over 65 years of age	Kochlik, B., (2019) [29]
ADMA	Plasma	Increased in frailty	Fried	Spain	2014	Group of 1287 people (552 men and 735 women), mean age of 74.4 years, of which 107 were frail, 542 pre-frail, and 638 not frail. In the group of frail people, ADMA presented higher values	Alonso-Bouzón, C. et al. (2014) [154]

**Table 7:** Biomarkers associated with hepatic and renal metabolism.

BIOMARKER	SAMPLE	CUT VALUES (FRAIL—NOT FRAIL)	FRAILTY SCALE	COUNTRY	YEAR	GROUP DESCRIPTION	REFERENCE
Uric acid (mg/dL)	Serum	5.49 ± 1.29 (Frail) 5.00 ± 1.06 (Pre-frail) 5.25 ± 1.23 (Not Frail)	Fried	Japan	2017	Group of 140 outpatients with probable Alzheimer’s disease, 65 years and older	Namioka, N. et al. (2017) [78]
Urate	Serum	Decreased in frailty	Edmonton Frail Scale (EFS)	Japan	2020	Group of 19 participants, mean age 84.2 ± 6.9 years, 7 men and 12 women, of which 9 were diagnosed as frail (age 88.2 ± 6.8 years) and 10 as not frail (age 80.5 ± 4.7 years)	Kameda, M., et al. (2020) [86]
Albumin (g/dL)	Serum	3.92 ± 0.31 (Frail) 3.96 ± 0.29 (Pre-frail) 4.06 ± 0.26 (Not Frail)	Fried	Japan	2017	Group of 140 outpatients with probable Alzheimer’s disease, 65 years and older	Namioka, N. et al. (2017) [78]
Bilirubin (mg/dL)	Serum	0.54 ± 0.18 (Frail) 0.60 ± 0.21 (Pre-frail) 0.66 ± 0.22 (Not frail)	Fried	Japan	2017	Group of 140 outpatients with probable Alzheimer’s disease, 65 years and older	Namioka, N. et al. (2017) [78]
Transferrin (ng/mL)	Plasma	58.3 ± 10.2 (Frail) 43.4 ± 11.4 (Not Frail)	Fried	United States	2013	Group of 65 people over 65 years old, *n* = 12 frail *n* = 22 not frail	Darvin, K. et al., 2013 [107]
Cortisol (µg/dL)	Serum	Increased in frail older people	Fried	Spain	2019	Study group of 252 elderly people (82 men and 170 women) from Spain, whose age range was 65–102 years	Marcos-Perez, D. (2019) [162]

**Table 8:** Biomarkers associated with DNA, RNA and miRNA.

BIOMARKER	SAMPLE	CUT VALUES (FRAIL—NOT FRAIL)	FRAILTY SCALE	COUNTRY	YEAR	GROUP DESCRIPTION	REFERENCE
miR-10a-3p	Plasma	Increased in frailty	Fried	United States	2018	Young people (ages 23–35 years) and people older than 65 years were recruited (the latter were classified as frail or not frail according to Fried’s criteria).	Ipson, B.R., et al. (2018) [167]
miR-92a-3p	Plasma	Increased in frailty	Fried	United States	2018	Young people (ages 23–35 years) and people older than 65 years were recruited (the latter were classified as frail or not frail according to Fried’s criteria)	Ipson, B.R., et al. (2018) [167]
miR-185–3p	Plasma	Increased in frailty	Fried	United States	2018	Young people (ages 23–35 years) and people older than 65 years were recruited (the latter were classified as frail or not frail according to Fried’s criteria)	Ipson, B.R., et al. (2018) [167]
miR-194–5p	Plasma	Increased in frailty	Fried	United States	2018	Young people (ages 23–35 years) and people older than 65 years were recruited (the latter were classified as frail or not frail according to Fried’s criteria)	Ipson, B.R., et al. (2018) [167]
miR-326	Plasma	Increased in frailty	Fried	United States	2018	Young people (ages 23–35 years) and people older than 65 years were recruited (the latter were classified as frail or not frail according to Fried’s criteria)	Ipson, B.R., et al. (2018) [167]
miR-532–5p	Plasma	Increased in frailty	Fried	United States	2018	Young people (ages 23–35 years) and people older than 65 years were recruited (the latter were classified as frail or not frail according to Fried’s criteria)	Ipson, B.R., et al. (2018) [167]
miR-576–5p	Plasma	Increased in frailty	Fried	United States	2018	Young people (ages 23–35 years) and people older than 65 years were recruited (the latter were classified as frail or not frail according to Fried’s criteria)	Ipson, B.R., et al. (2018) [167]
miR-760	Plasma	Increased in frailty	Fried	United States	2018	Young people (ages 23–35 years) and people older than 65 years were recruited (the latter were classified as frail or not frail according to Fried’s criteria)	Ipson, B.R., et al. (2018) [167]

## Data Availability

Not applicable.
